# Clinical outcomes during pregnancy and the postpartum period in women with myasthenia gravis and their newborns

**DOI:** 10.1055/s-0046-1829031

**Published:** 2026-10-09

**Authors:** Amanda Vitória Freire de Oliveira, Ana Flávia Andrade Lemos, Mariana Ribeiro Pereira, Rodrigo Siqueira Soares Frezatti, André Cleriston José dos Santos, Claudia Ferreira da Rosa Sobreira, Wilson Marques Junior, Pedro José Tomaselli

**Affiliations:** 1 Universidade de São Paulo, Faculdade de Medicina de Ribeirão Preto, Departamento de Neurociências e Ciências do Comportamento, Ribeirão Preto SP, Brazil.

**Keywords:** Myasthenia Gravis, Pregnancy, Pregnancy Complications, Pregnancy Outcome, Infant, Newborn, Diseases, Autoantibodies, Brazil

## Abstract

**Background:**

Myasthenia gravis (MG) is an autoimmune disease of the neuromuscular junction associated with increased maternal and fetal risks during pregnancy and the puerperal period.

**Objective:**

To describe the longitudinal course of MG, maternal and neonatal outcomes, and the occurrence of transient neonatal myasthenia gravis (TNMG) from the pregestational period until 12 months postpartum in pregnancies complicated by MG.

**Methods:**

A retrospective, longitudinal case-series study of pregnancies in women with MG who were followed at a single tertiary center for neuromuscular disorders. Disease severity was assessed using the MG Foundation of America Clinical Classification (MGFA)and MG-Activities of Daily Living (MG-ADL) scale. Treatments, delivery mode, postpartum exacerbations, ventilatory support and TNMG in newborns were collected using a standardized proforma. Longitudinal MG-ADL scores were analyzed using generalized estimating equations.

**Results:**

Twenty-four pregnant women with MG were included. Patients had juvenile-onset ( *n* = 12, 50%) or early-onset ( *n* = 12, 50%) MG, which was mostly generalized ( *n* = 20, 83%). Anti-AChR antibodies were positive in 71% ( *n* = 17) and anti-MuSK antibodies in 4% ( *n* = 1). Pyridostigmine was the main therapy; 8% of patients ( *n* = 2) experienced exacerbations during pregnancy. Cesarean delivery occurred in 60% ( *n* = 12), primarily for obstetric indications. The TNMG occurred in 15% of newborns (3 out of 20), and all fully recovered. The MG-ADL scores increased during the first and third trimesters, during the postpartum period, and at 12 months postpartum. Third-trimester MG-ADL scores ≥ 4 were associated with the occurrence of TNMG. No maternal or neonatal deaths occurred.

**Conclusion:**

Pregnancy in MG was characterized by variable disease activity, with late gestation and the postpartum period representing clinically relevant periods of vulnerability to maternal exacerbations and neonatal involvement. Increased third-trimester MG-ADL scores, particularly among antibody-positive mothers, were associated with TNMG and postpartum exacerbations, supporting close monitoring during these periods.

## INTRODUCTION

Myasthenia gravis (MG) is an autoimmune disease mediated by antibodies directed against components of the postsynaptic membrane of the neuromuscular junction (NMJ) and is considered a prototypical antibody-mediated neurological disorder because, in most cases, its pathophysiology is directly driven by well-characterized pathogenic autoantibodies with a clear and reproducible link to clinical manifestations. The global prevalence of MG has increased over recent decades and is currently estimated at 100 to 350 cases per million individuals. 1 The disease affects women approximately twice as often as men, particularly among those with onset before 50 years of age. In this subgroup, women most commonly present during the second and third decades of life, with female-to-male ratios reported to reach up to 3:1 in some cohorts, 2 3 4 overlapping with peak reproductive years.

The MG exposes pregnant women to an increased risk of maternal and fetal complications. The clinical course of MG during pregnancy is highly variable. Several studies have reported that disease exacerbations are more frequent during the first trimester and the postpartum period, 3 5 6 whereas symptomatic improvement has been observed during the second and third trimesters, likely reflecting physiological immunomodulation occurring at different stages of pregnancy. This immunomodulation is characterized by dynamic shifts in maternal immune responses, including alterations in T-cell balance and humoral immunity, which promote fetal tolerance while variably influencing autoimmune disease activity. 5 7 8

Smooth muscle function is not affected in MG; therefore, the mode of delivery is determined primarily by obstetric indications. However, the second stage of labor relies on sustained voluntary contractions of striated muscles, and assisted vaginal delivery may be required to reduce maternal expulsive effort and fatigue. 5

Transient neonatal myasthenia gravis (TNMG) is a well-recognized complication resulting from the transplacental transfer of maternal autoantibodies, leading to a self-limited myasthenic syndrome in the newborn. The reported incidence of TNMG varies widely, typically ranging from 10% to 15%. 7 9

Evidence regarding the interaction between pregnancy and MG remains limited. Uncertainty persists concerning disease behavior, optimal therapeutic management, and maternal–fetal outcomes, making counseling and clinical decision-making challenging for both patients and healthcare professionals. Data from Brazil are particularly scarce, with only one retrospective cohort study, reported by Ducci et al., providing information on pregnancy outcomes in women with MG. 10 Additional real-world studies may contribute to a better understanding of maternal disease course, obstetric outcomes, and neonatal complications across different clinical settings. The present study aimed to describe the clinical course and outcomes of pregnancy and the puerperium in women with MG, as well as neonatal outcomes in their offspring in a Brazilian population.

## METHODS

### **Study design and patient selection**

Women with confirmed MG who were followed at the Neuromuscular Disorders outpatient clinic of Hospital das Clínicas, Faculdade de Medicina de Ribeirão Preto, Universidade de São Paulo (HCFMRP-USP) and who had at least one pregnancy during the study period were included.

Clinical and serological data were retrospectively and longitudinally collected using a standardized proforma. The study was approved by the local Ethics Committee (protocol no. 87585824.8.0000.5440). Definitive cases were defined by the presence of a typical clinical presentation consistent with international consensus guidelines, characterized by fluctuating and fatigable weakness of voluntary muscles, together with neurophysiological evidence of neuromuscular junction dysfunction, defined as a decrement of greater than 10% in compound muscle action potential amplitude during low-frequency repetitive nerve stimulation in an affected muscle or abnormal single-fiber electromyography, or a positive antibody test or a clear clinical response to immunotherapy in seronegative patients. 11

Patients who expressed the desire to conceive during routine neurological follow-up received counseling regarding pregnancy-related risks and the use of immunosuppressive therapy. In some clinically stable patients, azathioprine had been discontinued before conception, either following medical advice or by patient preference, and treatment was maintained with pyridostigmine alone whenever possible. Patients previously treated with rituximab had received their last infusion at least 6 months before pregnancy onset.

### **Patient follow-up**

Functional status was assessed using the Myasthenia Gravis–Activities of Daily Living (MG-ADL) scale during the pregestational period (defined as the last clinical assessment before pregnancy), throughout each trimester of pregnancy, and during postpartum follow-up, including the first available postdelivery visit (< 30 days) and approximately 12 months after delivery. Treatment regimens were systematically recorded at the same time points. Clinical assessments were performed during routinely scheduled outpatient follow-up visits throughout pregnancy. For each trimester, data from the assessment closest to the corresponding gestational period were used for analysis. Clinical exacerbation was defined as worsening of MG symptoms requiring acute treatment with intravenous immunoglobulin (IVIg). Myasthenic crisis was defined as respiratory deterioration requiring ventilatory support. Episodes of clinical exacerbation and myasthenic crisis were assessed during pregnancy and the postpartum period based on clinical documentation in the medical records. The need for ventilatory support was recorded separately. Transient neonatal myasthenia gravis was diagnosed based on clinical signs, including generalized hypotonia, sucking difficulties, weak cry, and respiratory distress in the newborn.

### **Statistical analysis**

Descriptive statistics were used to summarize demographic, clinical, serological, and treatment-related characteristics. Continuous variables were expressed as mean ± standard deviation (SD) or median and interquartile range (IQR) values, as appropriate, whereas categorical variables were presented as frequencies and percentages. The MG-ADL scores from the pregestational period through 12 months postpartum were analyzed using generalized estimating equations (GEEs) with a negative binomial model and an unstructured correlation matrix, using prepregnancy scores as the reference category. Results are presented as odds ratios (ORs) with 95%CIs and corresponding *p* -values. Exploratory subgroup analyses were performed to evaluate associations between clinical and treatment-related variables and the occurrence of postpartum exacerbations or TNMG. Variables included age at onset (juvenile vs adult onset), clinical phenotype (ocular vs generalized MG), serological status (anti-AChR positive vs anti-AChR negative), treatment category (corticosteroid-containing vs noncorticosteroid regimens), third-trimester MG-ADL score (≥ 4 versus < 4), and third-trimester treatment with IVIg and/or prednisone. A post hoc exploratory analysis was also performed in the subgroup of mothers whose newborns developed TNMG. Comparisons of categorical variables between groups were performed using the chi-squared (χ ^2^ ) test or Fisher's exact test, as appropriate. Due to the small sample size, inferential analyses were considered exploratory and interpreted with caution. All statistical tests were 2-sided and statistical significance was set at *p* -value < 0.05. Analyses were performed using the IBM SPSS Statistics for Windows (IBM Corp.) software, version 20.0.

## RESULTS

Between 2005 and 2025, a total of 332 women with confirmed MG were followed at our institution. Of these, 217 (65%) were of reproductive age (15-49 years). Twenty-four women experienced a total of 24 pregnancies during the study period and were included in the analysis. Among the included women, age at disease onset ranged from 8 to 34 (mean: 18.2 ± 6.3; median 17.5 [15.3–21]) years. A total of 12 patients (50%) had juvenile-onset disease, and 12 (50%) had early-onset disease. At diagnosis, 20 patients (83%) presented with generalized MG, including 5 with predominant bulbar involvement, whereas 4 (17%) had ocular MG. Anti-AChR antibodies were tested by ELISA in all patients and were positive in 17 of 24 patients (71%). Anti-MuSK antibodies were tested in four patients and were positive in one patient with early-onset generalized MG. The remaining patients were not tested for anti-MuSK antibodies because this assay was not available at our institution during part of the study period. Seventeen patients had undergone thymectomy before pregnancy, including one due to thymoma ( Table 1 ).

**Table 1:** Baseline clinical characteristics, obstetric outcomes, and treatment during pregnancy

Variable	
Number of pregnancies	24
Mean age at MG onset	18.2 ± 6.3
	**n (%)**
Juvenile-onset MG	12 (50%)
Early-onset MG	12 (50%)
Generalized MG	20 (83%)
Ocular MG	4 (17%)
Anti-AChR positive	17 (71%)
Anti-MuSK positive	1/4 (25% of tested patients)
Thymectomy	17 (71%)
Caesarean delivery	12/20 (60%)
Vaginal delivery	8/20 (40%)
TNMG	3/20 (15%)
Preterm delivery (< 37 weeks)	1/20 (5%)
Clinical exacerbation during pregnancy	2/23 (9%)
**Treatment during pregnancy**	
Pyridostigmine alone (1st trimester)	17/24 (71%)
Pyridostigmine alone (2nd trimester)	18/23 (78%)
Pyridostigmine alone (3rd trimester)	16/23 (70%)
IVIg during pregnancy	2/24 (8%)
Azathioprine exposure at pregnancy recognition	5/24 (21%)
Rituximab exposure within 6 months before conception	2/24 (8%)

A total of 19 patients were followed for at least 12 months after delivery. One patient was lost to follow-up during the first trimester, three delivered at other institutions and did not attend the postpartum visit, and one did not attend the 12-month follow-up evaluation ( Figure 1 ). Among the five patients lost to follow-up, two had relocated to different centers, one reported being asymptomatic and did not return for evaluation, and two could not be contacted.

**Figure 1:**
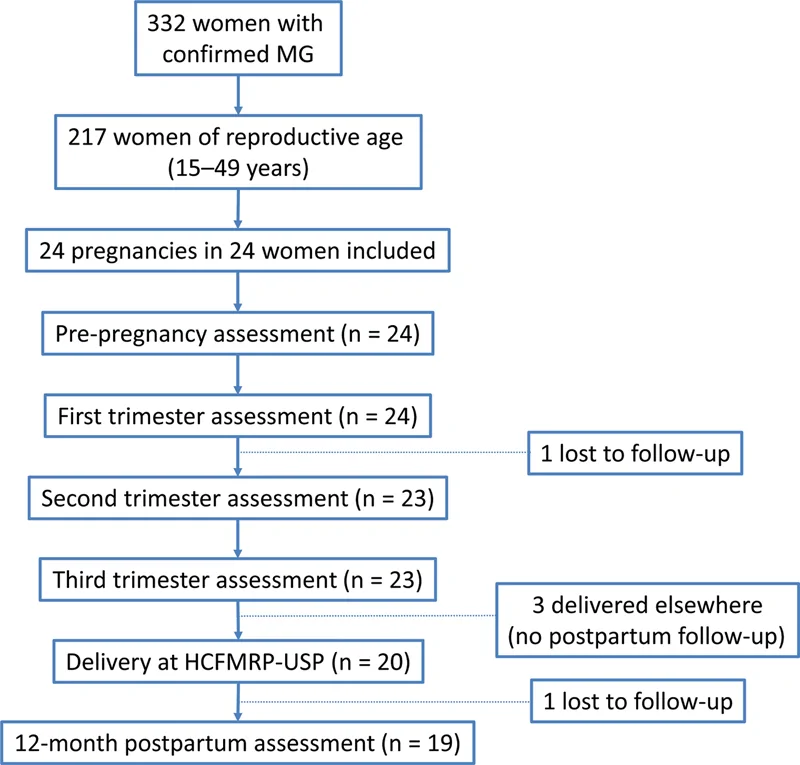
Flowchart of patient selection and follow-up during pregnancy and the postpartum period.

In a subset of clinically stable patients, azathioprine had been discontinued before conception, either on medical advice or by patient preference, and treatment was maintained with pyridostigmine alone whenever possible. Two patients had received rituximab within 6 months before pregnancy onset. At the time pregnancy was recognized, five patients were receiving azathioprine, two had previously received rituximab, and one was being treated with monthly IVIg combined with prednisone. During gestation, pyridostigmine was the mainstay of treatment across all trimesters. In the first trimester, 17 women (71%) were managed with pyridostigmine alone, 2 required monthly IVIg, 1 of whom also received prednisone, while another patient required prednisone combined with pyridostigmine only. Three women were not receiving medication, and one patient was lost to follow-up during this period. Similar treatment patterns were observed during the second and third trimesters, with prednisone added for one patient during the second trimester and another during the third trimester.

Two of 23 patients (9%) experienced clinical exacerbations during the third trimester ( Table 1 ), requiring IVIg therapy because of significant dysphagia and dyspnea. No patients developed myasthenic crisis requiring ventilatory support, and no maternal deaths were recorded.

Twelve out of 20 women (60%) underwent cesarean section and 8 (40%) had vaginal delivery. Cesarean sections were performed due to obstetric indications, including 1 preterm delivery before 37 weeks of gestation. The TNMG occurred in 3 out of 20 newborns (15%) ( Table 1 ). Two of the three affected newborns required ventilatory support, and one required prolonged hospitalization. No neonatal deaths occurred, and all affected newborns fully recovered.

At the first postdelivery evaluation, 11 patients were receiving pyridostigmine alone; 3 were treated with azathioprine plus pyridostigmine; 3 with prednisone plus pyridostigmine; 2 with monthly IVIg, prednisone, and pyridostigmine; 1 with monthly IVIg plus pyridostigmine; and 2 were not receiving medication. Two patients had received rituximab within 6 months before conception and were clinically stable at conception (MG-ADL scores of 0 and 2). One patient was anti-MuSK positive and the other was anti-AChR positive. During the first trimester, the anti-AChR-positive patient experienced clinical worsening, with the MG-ADL score increasing from 2 to 5, whereas the anti-MuSK-positive patient remained clinically stable. During the second and third trimesters, symptoms remained overall stable or improved. One patient remained stable at MG-ADL score of 2 throughout the second and third trimesters, whereas the other improved to MG-ADL score of 2 during the second trimester and subsequently increased to 4 during the third trimester. At the first postpartum assessment, both patients had lower MG-ADL scores (1 and 2); however, at the 12-month postpartum follow-up, their MG-ADL scores had increased again (5 and 4, respectively). Two additional patients treated with pyridostigmine alone also demonstrated increased MG-ADL scores during long-term postpartum follow-up despite remaining clinically stable throughout pregnancy and the early postpartum period, with MG-ADL scores reaching 2 and 8 at 12 months after delivery.

### **Inferential statistical analysis**

The MG-ADL scores showed a significant increase compared with the pregestational period. Higher MG-ADL scores were observed during the first trimester (OR = 2.67; 95%CI: 1.05–6.74; *p* = 0.04), third trimester (OR = 3.62; 95%C:I 1.62–8.11; *p* = 0.002), first postpartum evaluation (OR = 3.60; 95%CI: 1.53–8.51; *p* = 0.003), and at 12-month follow-up (OR = 3.00; 95%CI: 1.25–7.20; *p* = 0.01) ( Table 2 ).

**Table 2:** MG-ADL scores across pregnancy and postpartum, with GEE estimates (reference: prepregnancy)

Time point	n	Mean	SD	Median (range)	β (SE)	OR	95%CI	*p* -value
**Prepregnancy**	24	0.50	0.98	0 (0–4)	0 (Ref)	1 (Ref)	–	–
First trimester	24	1.33	2.08	0 (0–6)	0.98 (0.47)	2.67	1.05–6.74	0.04
Second trimester	23	0.83	1.37	0 (0–5)	0.46 (0.49)	1.58	0.60–4.16	0.351
Third trimester	23	1.83	2.37	1 (0–8)	1.29 (0.41)	3.62	1.62–8.11	0.002
First postpartum visit	21	1.71	2.22	1 (0–8)	1.28 (0.44)	3.61	1.53–8.51	0.003
12-month follow-up	19	1.42	2.27	0 (0–8)	1.10 (0.45)	3.00	1.25–7.20	0.014

Abbreviations: GEE, generalized estimating equations; MG, myasthenia gravis; MG-ADL, myasthenia gravis activities of daily living; OR, odds ratio; SD, standard deviation; SE, standard error; TNMG, transient neonatal myasthenia gravis.

Note: Prepregnancy values were used as the reference category.

Exploratory analyses were performed to evaluate whether clinical features, serological status, or treatment-related variables were associated with postpartum exacerbations and TNMG. No significant associations were found for age of onset (juvenile-onset versus early-onset) ( *p* = 0.21), clinical classification (ocular or generalized) ( *p* = 0.59), serological status ( *p* = 0.52), or treatment prior to gestation ( *p* > 0.05). The use of IVIg and/or prednisone during the third trimester was significantly associated with postpartum exacerbations (χ ^2^ = 26.56; *p* = 0.022). Third-trimester MG-ADL scores ≥ 4 were associated with an increased risk of TNMG ( *p* = 0.04). Among AChR-antibody-positive mothers, the mean absolute change in MG-ADL score was 2.5 points in those whose newborns developed TNMG, compared with 0.57 points in those whose newborns did not.

## DISCUSSION

Myasthenia gravis is a rare neuromuscular disorder that can significantly affect pregnancy, which is generally considered a state of physiological immunosuppression. There is evidence, although not yet fully elucidated, that polymorphonuclear leukocyte chemotaxis and adherence functions are reduced beginning in the second trimester and remain suppressed throughout the remainder of pregnancy. This reduction in leukocyte activity may partially explain the clinical improvement observed in certain autoimmune disorders. 3 12 Conversely, it is well established that some autoimmune diseases may exacerbate during pregnancy. 12 Although exacerbations most commonly occur in the first trimester and the postpartum period, the clinical course of MG during pregnancy remains highly variable.

In this Brazilian cohort of pregnant women with MG, we evaluated maternal and neonatal outcomes. All participants had juvenile-onset or early-onset disease, and most patients were clinically stable at conception. Nonsteroidal immunosuppressive therapies were discontinued upon confirmation of pregnancy or before conception in planned pregnancies. Pyridostigmine remained the cornerstone of symptomatic treatment throughout gestation.

The MG-ADL scores increased consistently during the first and third trimesters, as well as during the postpartum period and at 12-month follow-up, compared with the pregestational baseline. The deterioration observed in the third trimester is consistent with previous reports describing worsening in up to one-third of pregnancies, typically during the final four weeks of gestation and the puerperium. 13 In contrast, some studies have reported clinical deterioration during late pregnancy, including the third trimester. 5 14 In our cohort, this pattern was accompanied by an increased need for immunosuppressive therapy during pregnancy compared with baseline treatmentto achieve adequate symptom control.

During pregnancy, crisis or exacerbation requiring hospitalization occurred in 8.4% (2/23) of patients, both in the third trimester. Postpartum exacerbations occurred in 15% of cases, and one patient (5%) required a prolonged hospital stay. These findings are consistent with prior evidence suggesting an increased risk of myasthenic worsening in the immediate postpartum period, previously reported in approximately 14.8% of pregnancies. 15 Although 10 to 20% of patients with MG are expected to experience a myasthenic crisis and 12 to 22% to have at least one annual exacerbation, most women in our cohort had no prior history of exacerbation or crisis before pregnancy, supporting the potential vulnerability of the postpartum period in otherwise clinically stable patients. 16

Cesarean delivery was performed in 60% (12/20) of cases, exclusively for obstetric indications, a rate comparable to the overall cesarean delivery proportion in Brazil (56.44%) 17 and similar to rates reported in other middle-income settings, as well as in a previously published Brazilian cohort. 10 This finding is in line with current recommendations suggesting that mode of delivery in MG should be guided primarily by obstetric rather than neurological considerations. Reported vaginal delivery rates among patients with MG vary substantially (from 58–90%), likely reflecting institutional practices and patient characteristics rather than disease-related factors. 14 18

Serological findings were consistent with previous MG series, with anti–AChR antibodies detected in approximately 70% of cases and anti-MuSK antibodies in approximately 4%. 9 19 Although anti-MuSK status could not be fully assessed due to limited test availability, the overall serological profile did not appear to influence obstetric outcomes, postpartum exacerbations, or neonatal complications.

The TNMG was observed in 15% of newborns, and all affected infants recovered completely. This rate is comparable to that reported in other studies, including a previously published Brazilian cohort and larger case series, 10 14 15 16 and it is also consistent with the known occurrence of transient neonatal myasthenia gravis in exposed neonates. The TNMG results from the transplacental transfer of maternal autoantibodies, predominantly anti-AChR antibodies. 9 Although passive transfer is common, fewer than 20% of exposed neonates develop clinical manifestations. 7 9

Notably, mothers with anti-AChR antibodies whose newborns developed TNMG showed greater MG-ADL score variation during the third trimester compared with the pregestational period and the first trimester. Higher MG-ADL scores (≥ 4) in late pregnancy were associated with increased odds of TNMG, suggesting that third-trimester disease worsening may be associated with neonatal involvement and may warrant closer peripartum monitoring and preparedness for specialized neonatal care. Although antibody titers were not available, some studies have reported an association between higher anti-AChR levels and increased TNMG risk. 20

Exploratory regression and correlation analyses did not identify significant associations between postpartum exacerbations or TNMG and age at onset, clinical phenotype, or pregestational treatment. Treatment with IVIg and/or prednisone during the third trimester was associated with postpartum exacerbations, highlighting the potential relevance of disease severity and treatment requirements in late pregnancy.

This study has several limitations, including its retrospective design, small sample size, and incomplete serological and neurophysiological data in a subset of patients. Rare outcomes such as postpartum crisis or TNMG may not have been fully captured due to the limited sample size.

These findings are consistent with the previously published Brazilian cohort, 10 which also reported similar patterns of maternal disease fluctuation during pregnancy, rates of cesarean delivery predominantly driven by obstetric indications, and comparable frequency of transient neonatal myasthenia gravis. Together, these data suggest a consistent clinical profile across Brazilian centers, despite differences in study design and patient selection.

Taken together, these findings suggest that pregnancy in MG is characterized by variable disease activity, with late gestation and the postpartum period representing clinically relevant phases of vulnerability for both maternal exacerbation and neonatal involvement. This study provides original data from a Brazilian cohort and supports the use of MG-ADL as a practical tool for longitudinal monitoring and clinical assessment in pregnancy.

In summary, pregnancy in women with MG is characterized by variable disease activity, with late gestation and the postpartum period representing clinically relevant phases of vulnerability for maternal exacerbations and neonatal involvement. Our data suggest that positive antibody status and greater worsening of MG-ADL scores between the pregestational period and the third trimester were associated with TNMG and peripartum exacerbation. These findings support individualized management strategies and emphasize the importance of multidisciplinary care throughout pregnancy and the postpartum period.
